# Grading reflective essays: the reliability of a newly developed tool- GRE-9

**DOI:** 10.1186/s12909-020-02213-2

**Published:** 2020-09-25

**Authors:** Nisrine N. Makarem, Basem R. Saab, Grace Maalouf, Umayya Musharafieh, Fadila Naji, Diana Rahme, Dayana Brome

**Affiliations:** 1 Department of Family Medicine, American University of Beirut-Medical Center, Riad El-Solh, P. O Box 11-0236, Beirut, 1107 2020 Lebanon; 2 Department of Psychology, Haigazian University, Rue Mexique, Kantari, Riad el Solh, P.O.Box: 11-1748, Beirut, 11072090 Lebanon

**Keywords:** Reflective writing, Reflective articles, Grading tools, Reflection

## Abstract

**Background:**

The main objective of this study is the development of a short reliable easy-to-use assessment tool in the aim of providing feedback to the reflective writings of medical students and residents.

**Methods:**

This study took place in a major tertiary academic medical center in Beirut, Lebanon. Seventy-seven reflective essays written by 18 residents in the department of Family Medicine at the American University of Beirut Medical Center (AUBMC) were graded by 3 raters using the newly developed scale to assess the scale reliability. Following a comprehensive search and analysis of the literature, and based on their experience in reflective grading, the authors developed a concise 9-item scale to grade reflective essays through repeated cycles of development and analysis as well as the determination of the inter-rater reliability (IRR) using intra-class correlation coefficients (ICC) and Krippendorff’s Alpha.

**Results:**

The inter-rater reliability of the new scale ranges from moderate to substantial with ICC of 0.78, 95% CI 0.64–0.86, *p* < 0.01 and Krippendorff’s Alpha was 0.49.

**Conclusions:**

The newly developed scale, GRE-9, is a short, concise, easy-to-use reliable grading tool for reflective essays that has demonstrated moderate to substantial inter-rater reliability. This will enable raters to objectively grade reflective essays and provide informed feedback to residents and students.

## Background

Reflective practice within medical education is considered an essential aspect of lifelong self-directed learning becoming a crucial element of the medical program at all its levels aiming towards a competence-based curriculum [1]. The idea of reflective practice was first established by Schon in 1987 and characterized by three stages: awareness of thoughts and feelings, critical analysis of a condition, and development of a new viewpoint of the situation [2]. Hence, it follows that reflection allows the development and integration of new knowledge into practice leading to the core experience of greater professional competence [3]. A growing body of research with regard to reflection in the medical education literature highlighted the relationship between reflective capacity and the enhancement of physician competence [4–7].

Given the beneficial consequences of reflection [8], medical educators have sought to explore a variety of methods for fostering and assessing reflection in learners, ranging from one-to-one mentoring [9] to guided discussions [10], digital approaches like video cases [11] and written methods like reflective portfolios, journal and essay writings [9, 12]. Reflective writing was reported to be one of the most extensively and widely used forms of reflective teaching in medical education [13, 14]. Reflective capacity within these reflective writing exercises can be assessed through various qualitative and quantitative tools [15]. Despite the presence of diverse methods, there is still a lack of best practices [15]. With the proliferation of reflective writing in promoting and assessing reflection [16], the need for a valid and reliable evaluative tool that can be effectively applied to assess students’ levels of reflection was strongly called for [17].

Existing modalities of reflection evaluation identified in the literature include scales (“paper and pencil” forms with responses scored by respondents), qualitative analysis including thematic coding and more elaborate analysis moving beyond themes into models, and analytical instructional rubrics (theory-based delineation of dimensions or levels of an assessed construct) [17]. Given that quantitative tools are primarily used in research for curriculum improvement and to guide feedback in reflection practice, analytic rubric assessment tools are extensively chosen in assessing reflective writings [15]. These rubrics provide a precise data with multiple reflective dimensions that educators can reference to when presenting feedback [15] leading to a remarkable improvement in reflective capacity of students [18].

These analytic rubric models are based on theoretical frameworks [17]. Of the major theoretical foundations is Mezirow’s (1991) reflective taxonomy which explains reflection as a basis for the transformative process in learning [19]. Theoretical foundations for such analytic rubrics also include Schon’s (1983) [20] focus on progression from knowing-in-action, to reflection-in-action, experimentation and reflection-on-action (post-experience reflection) as well as Boud and colleagues’ (1985) accentuation on feelings in the reflective process [21]. For instance, Kember et al. [22] proposed four methodological requirements for the measurement reflection tool. These incorporated the focus and direct assessment of reflection, the avoidance of construct-irrelevant variances in the conceptualization of the reflection themes, the specified detail of the method and procedure to ensure transparency and replication and an obtainment of an appropriate testing of reliability. Their methodological requirements were mainly based on Mezirow’s (1991) conceptualization of reflection [12] and they incorporated four categories: habitual action, understanding, reflection and critical reflection.

Notable limitations and challenges with regard to the available coding systems and tools used in the analysis and assessment of reflective writings are documented in the literature [17]. Some published rubrics for reflective narrative analysis are limited in scope as well as in validity [23–25]. Others lack a reliable structured worksheet that assesses levels of reflection, are relatively difficult, and are low on reliability outcomes [16, 26]. Quantitative evidence supporting the psychometric properties of some of these reflective assessment tools is limited [5], with evidence regarding the inter-rater reliability of those tools still at its preliminary levels [17, 22]. Given these challenges and limitations, and the notion that reflection is hard to measure and assess directly [12], it becomes imperative to develop simpler tools that are short, concise, include well-defined descriptors, and are easily accessible for analysis and interpretation with high level of objectivity. Despite the implementation of rater training efforts for reflective writings of students, rater variability in scoring remains to be a source of concern [27, 28]. Given that students’ approaches to learning might be affected by the type of assessment strategy used [29, 30], unreliable assessment strategies can lead to unfair results. Hence, designing a reliable assessment tool is needed to decrease the incidence of rater variability [6].

Consequently, this study serves as a step in filling this research gap by developing an empirically tested and concise new reflective writing assessment tool and exploring its inter-rater reliability with the aim of establishing a reliable measure of reflective writing.

## Methodology

### Overview of study design

#### Instruments and procedures

For the past several years, the reflective essays written by Family Medicine residents during 3 years of training (between June 2014 and May 2017) were graded by their advisors using a simple grading scale. This scale consists of a simple guide on what the advisor should consider while grading (Additional file 1). Throughout the past several years, concerns were raised by numerous faculty members as to the ambiguity of this scale and the lack of standardization of grading. Hence, the aim to develop a new concise and reliable tool to evaluate reflective essays written by residents at the AUBMC was established. Since the essays are part of a routine formative assessment activity, the study was qualified as exempt from the Institutional Review Board (IRB) at the AUBMC.

#### Development of the scale

In an effort to improve the reflective essays grading process at AUBMC, a new scale was developed by faculty members at the Department of Family Medicine. The development of the scale began with an explorative literature review; three Family Medicine physicians versed in the field of medical teaching, curriculum development, as well as reflective writing assessment screened and discussed an initial pool of items for relevance. The literature review included existing theoretical models of reflection, reflective writing pedagogy, elements of reflective practice, and existing assessment modalities in health professions education.

The analytical instructional rubric model was chosen as an evaluative model for the development of our reflective tool. The analytic rubric model outlines diverse reflective dimension levels and assessment criteria, defines benchmark for each of these levels, yields quantitative scores and is used for formative and summative purposes [17, 31, 32]. Analytic-type rubrics also provide total and domain specific scores; in this case allowing educators to identify the global and the domain specific deficits in the reflective skills of the learners and provide accordingly the specific and constructive feedbacks [15].

In the first cycle of constructing the initial reflection rubric, the rubric was based on a comprehensive analysis of relevant theoretical models of reflection as well as existing reflection assessment measures [33]. After taking into account a wide range of elements, an agreement was reached in incorporating the levels of reflection which were associated with criteria based on theories of Mezirow [19], Schon [2] and Boud and colleagues [21]. More specifically, the framework scheme of REFLECT rubric, a rigorously developed and a theory-informed analytic rubric was the starting point for item selection [17]. The REFLECT rubric provides a comprehensive, logical and credible theoretical principles based on Mezirow’s [19] conceptualization of the different levels of reflection, Schon’s [2] theory about the reflective practitioner as well as Boud’s and colleagues [21] reflective analysis. The four levels of reflective capacity of the REFLECT rubric incorporate: (1) habitual action (2) thoughtful action (3) reflection and (4) critical reflection which incorporates transformative learning (new understanding) and confirmatory learning (confirming one’s frames of reference or meaning structure). These four reflective levels include core processes that assimilate writing spectrum, presence, recognize “disorienting” dilemmas, recognize critical analysis of assumptions, attend to emotions, and derive meaning features [17]. On the basis of these theoretical levels of reflection, the process of developing our analytical instructional rubric commenced on the basis of an accepted methodology, listing of criteria, designating quality levels, creating a rubric draft with further revisions and refining of the draft based on targeting each of the four levels. The items of the Groningen Reflection Ability Scale (GRAS) formed further theoretical frameworks for the newly developed scale. These included three emerged thematic factors of personal reflection: self-reflection, empathetic reflection and reflective communication of the existing reflection assessment measure [1]. Items of this measure are grounded in reflection literature, and cover three substantive aspects of personal reflection in the context of medical practice and education: Self-reflection, Empathetic Reflection; and Reflective Communication. Self-reflection focuses on the exploration and appraisal of experience and forms a basis to the individual to frame or reframe one’s thoughts, feelings, beliefs, norms or methods. Empathetic reflection focuses on contextual understanding and appraisal when engaging in empathetic placement and when thinking about the position of others, such as patients and colleagues. As for reflective communication, it allows the handling of feedback and discussion, as well as dealing with interpersonal differences and taking responsibility of one’s statements and actions [1].

The first cycle yielded the first draft of the newly developed scale that consisted of 11 items. This scale then underwent a second cycle that included the previous 3 physicians along with 3 additional physicians. Each physician was asked to use the new 11-item scale to grade 3 reflective essays and to provide feedback related to the objectivity of the scale, the time needed to grade using the scale, the clarity of the items, and the ease of use. After several meetings and discussions between the faculty members, some items were reformulated and 2 items were dropped namely “comments on response” and “critical thinking”. The remaining sample items match with the four levels of the reflective capacity of the REFLECT rubric and the three thematic factors of personal reflection of the GRAS. Table 1 shows the matching comparison.

**Table 1:** Matching of the GRE-9 items with the REFLECT Rubric reflective evaluation levels and Groningen Reflection Ability Scale’s (GRAS) theoretical facets

GRE-9 items	REFLECT Rubric Criterion/ Axis	GRAS reflective theoretical facets
1. What happened?	Description of conflict or disorienting dilemma	Self-reflection criterion^a^
2. What is special about this event?		
3. Feelings when it happened	Attending to emotions	Self-reflection criterion^a^
4. What was the outcome for the concerned?	Description of conflict or disorienting dilemma	Empathetic reflection criterion^b^
5. Understanding of the event	Description of conflict or disorienting dilemma	Self-reflection criterion^a^
6. Congruence of actions and beliefs	Analysis and meaning making	Self-reflection criterion^a^
7. New thoughts and feelings after reflection	Analysis and meaning making	Empathetic reflection criterion^b^
8. Reference to old experience and others*	Critical reflection/ confirmatory learning	Reflective communication criterion^c^
9. How this incident will affect future role	Critical reflection/ Transformative learning	Reflective communication criterion^c^

^a^The introspective aspect of personal reflection: The careful exploration and appraisal of experience, as a prerequisite for framing or reframing one's thoughts, feelings, beliefs, norms or methods

^b^The social, inter-subjective extension of self-reflection: Contextual understanding and appraisal, i.e. empathetic placement in and thinking about the position of others, such as patients and colleagues

^c^The behavioral expression of both self-reflection and empathetic reflection, for example the handling of feedback or a dialogue, or dealing with interpersonal differences

*Reference to others is unique to GRE-9

Adapted from: Wald HS, Borkan JM, Taylor JS, Anthony D, Reis SP. Fostering and evaluating reflective capacity in medical education: developing the REFLECT rubric for assessing reflective writing. Acad Med. 2012;87:41-50.

Adapted from: Aukes LC, Geertsma J, Cohen-Schotanus J, Zwierstra RP, Slaets JPJ. The development of a scale to measure personal reflection in medical practice and education. Med Teach. 2007

A session was then conducted to decide on the standardization of the scoring where a rationale was presented and scoring discrepancies were resolved after which a scoring consensus was reached. The first 2 items of the scale, which are descriptive, are given a maximum grade of 1 whereas the rest, which are analytical, are given a maximum grade of 2. The maximum score is 16. The items are followed by a guide that clarifies each point with the aim of facilitating and standardizing the grading process. The scale referred to as the Grading Reflective Essays − 9 (GRE-9) from here on consists of 9 items. Table 2 includes scoring per item as well as the guidance for grading. Eighteen Family Medicine residents training in a four-year program at the AUBMC were asked to write reflective essays based on incidences from their medical practice. Papers were then randomly coded by the authors. Before applying the new scale, several meetings were held for the raters to discuss debatable points and to try to unify the grading system as much as possible. The raters were asked to read the entire essay and then fragment it into parts corresponding to each of the items assessed in the GRE-9. The presence and quality of each item criteria should then be assessed by giving a partial point if an item criterion has been mentioned but not in depth and a full score for critical reflection. All 3 raters were asked to provide an overall feedback on ease-of-use of the scale in terms of clarity of items and time needed to grade.

**Table 2:** Scoring of GRE-9 per item and guidance for grading

Item	Not attempted	Partial	Full
**1. What happened?** *Guidance: State the main features of the event: persons involved, timing, place, setting, how all persons concerned acted/behaved*			1
**2. What is special about this event?** *Guidance: State clearly the reason for choosing this event in particular. In-depth description of the dilemma, conflict, challenge posed by the event*			1
**3. Feelings when it happened** *Guidance: Describe personal thoughts and feelings while the event was happening, emotional insight and empathy*			2
**4. What was the outcome for the concerned?** *Guidance: Concerned include patient, significant others, health professionals, health system, society. Empathetic reflection*			2
**5. Understanding of the event** *Guidance: Express what was good and bad about the experience; interpretation of the situation at present with justifications (factors/ knowledge influencing judgment)*			2
**6. Congruence of actions and beliefs** *Guidance: Does the resident think he acted as per his beliefs? Was there anything that held him back from applying his beliefs? Reflection-on-action*			2
**7. New thoughts and feelings after reflection** *Guidance: Describe resident’s new thoughts and feelings after reflecting on the case. Making meaning and analysis of the thoughts and feelings*			2
**8. Reference to old experience and others** *Guidance: Compare to other situations, experience, others involved, and known events and facts, preferable to have reference*			2
**9. How this incident will affect future role** *Guidance: If it arose again, how would the resident act? How did this experience change the practice for the better? Transformative learning, development of new viewpoint of the situation*			2
**Total score**			16

#### Sample size for reliability assessment

Given that the sample size calculations for reliability assessment are based on testing for a statistical difference between moderate (i.e., 0.40) and high (i.e., 0.75) kappa values using alpha of 0.05 and beta error rates of 0.2, the estimated sample size would range from 77 to 28 based on the variability of trait prevalence between 10 and 50% [34]. Given that Kappa values are transferred to Krippendorff’s alpha [35], the sample size of 77 provides the needed power to detect a statistically significant Krippendorff’s alpha.

Since the minimally acceptable level of Krippendorff’s alpha is at least 0.40, then three raters per subject are required because increasing the number of raters beyond 3 has little effect on the power of hypothesis tests [34, 36]; as such, three raters were chosen to evaluate the reflective pieces.

Similarly, taking into consideration the calculation of intra-class correlation (ICC), the calculation of the minimum sample size to estimate the value of ICC was performed by using Power Analysis and formula for minimum sample size (n) estimation using the PASS software which is derived from previous studies [36, 37]. With three raters (k = 3) used, pre-specification of an acceptable reliability and an expected reliability of 0.0 and 0.2 respectively and a power set to be at least 80% and value of alpha set to be 0.05, the minimum sample size obtained through the formula is approximately 60 or 61 participants. Given that increasing the number of subjects is the more effective strategy for maximizing power [38] and given that the estimated sample size of 77 provides the needed power to detect a statistically significant Krippendorff’s alpha, 77 reflective pieces were used to calculate ICC as well.

Consequently, three family physicians, who were involved in the second cycle of the scale development, evaluated the 77 reflective essays. Rater1 is a full time faculty, has been in practice for 24 years, is a professor and is well versed in the area of assessing reflective capacity. The other two raters are part time faculty, one who has been in practice for 15 years (Rater 2) while the other for 5 years (Rater 3).

The three raters were asked to grade 3 essays at one time every other day. Each rater was asked to log the number of the essay and the grade assigned as well as the time needed to read and grade each essay. The essays were reviewed in the same order by all reviewers. Anonymity was assured through randomization of the essays into alphanumeric codes.

#### Statistical analysis

Descriptive statistics were used to quantify the level of higher order processing evident in each reflective piece. Interrater reliability was assessed for each level of cognitive processing as well as for the highest level of cognitive processing evident within each entry using ICC and Krippendorff’s alpha.

Among the six different equations or models for calculating the ICC, Model 2 was selected in this research study considering each element or entry of the reflective piece being assessed by all raters who are considered representative of a larger population of potential raters with the expectation that the results may be generalized to other raters with similar characteristics. More specifically, examining the inter-rater reliability of continuous variables, such as total score for the 9 questions for each of the three raters in the GRE-9 scale, the ICC including a two-way random model (ANOVA) with absolute agreement was used for calculation. Given that the aim was to generalize the reliability results to any raters who possess the same characteristics as the selected raters, a two-way random effects model was the appropriate model to use as it specifies that each subject (reflective essay by each student) is evaluated by the same set of *k* independent raters, who have been randomly sampled from a larger population of raters with similar characteristics [35]. Also, given that the absolute agreement concerns about the extent to which the raters provide the equal scores and implies that all raters match scores exactly [39, 40], the absolute agreement type was chosen to ensure greater precision. When establishing criteria to judge acceptable levels of reliability the authors used the criteria established by Landis and Koch [35] to judge the strength of reliability reported through the ICC with values of ICC < 0 representing poor agreement, 0.01–0.20 representing slight agreement, 0.21–0.40 representing fair agreement, 0.41–0.60 representing moderate agreement, 0.61–0.80 representing substantial agreement, and 0.81–1.00 representing almost perfect agreement and thus high reliability. With regard to Krippendorff’s alpha, when values range from 0 to 1, 0 is considered perfect disagreement and 1 is considered perfect agreement. According to Krippendorff [41], α ≥ .800 is the required value, and α ≥ .667 is considered an acceptable value. However, it was also reported that the cut-off value scores provided by Landis and Koch (1971) [35] can also be transferred to Krippendorff’s alpha [42].

## Results

The average score for the 3 raters is 10.03 ± 2.44 with the average of rater 1, 2 and 3 being 9.38 ± 2.94, 9.68 ± 2.39, and 11.04 ± 2.44 respectively. The average time needed to read and grade an essay of an average of 500 words was 4.5 min (SD = 2.0; range 2–12 min).

The obtained ICC was 0.78 indicating substantial agreement, 95% CI 0.64–0.86 (*F*(76, 152) = 5.781, *p* < 0.01). (Table 3).

**Table 3:** Intra-class correlation coefficients (ICC) of the 3 Raters across the GRE-9 items (*N* = 77)

	ICC (95%, CI)
Rater1 versus Rater2 Versus Rater3	0.78 [0.64–0.86]
Item 1	0.66 [0.51–0.77]
Item 2	−0.06 [−0.31–0.18]
Item 3	0.70 [0.58–0.81]
Item 4	0.60 [0.41–0.73]
Item 5	0.41 [0.14–0.60]
Item 6	0.52 [0.30–0.68]
Item 7	0.67 [0.51–0.78]
Item 8	0.52 [0.30–0.68]
Item 9	0.62 [0.45–0.75]

To examine the inter-rater reliability of ordinal variables for more than 2 raters, the Krippendorff’s Alpha was also calculated. Total agreement between the 3 raters was 0.49 (moderate). (Table 4).

**Table 4:** Krippendorff’s alpha of the 3 Raters across the GRE-9 items (*N* = 77)

	Krippendorff’s alpha (95%, CI)
Rater1 versus Rater2 Versus Rater3	0.49 [0.25–1.0]
Item 1	0.49 [−.25–1.00]
Item 2	− 0.17 [− 0.36–0.01]
Item 3	0.47 [0.36–0.57]
Item 4	0.29 [0.16–0.41]
Item 5	0.18 [0.06–0.31]
Item 6	0.25 [0.23–0.46]
Item 7	0.35 [0.23–0.46]
Item 8	0.31 [0.16–0.45]
Item 9	0.34 [0.22–0.45]

The 3 raters provided positive feedback on the usability of the GRE-9 as was inferred from the clarity of the items, the grades assigned to each item, the time needed to grade, and the elaborative guide provided with the scale that explains each individual item.

Excluding item 2, the ICC estimates and krippendorff’s alpha for each of the 9 items varied from 0.41 to 0.70, and 0.18 to 0.49, respectively (Tables 3 and 4). The lowest ICC value and krippendorff’s alpha were obtained for item 2 “What is special about this event- State clearly the reason for choosing this event in particular” (ICC = − 0.06, Krippendorff’s alpha = − 0.17,). Item 5 “Understanding of the event - Express what was good and bad about the experience; interpretation of the situation at present with justifications (factors/ knowledge influencing judgment)” showed a low agreement amongst the raters (ICC = 0.41, Krippendorff’s alpha = 0.18). Item 3 “Feelings when it happened - Describe personal thoughts and feelings of the resident while the event was happening” (attending to feelings) obtained the highest ICC (0.70) and the second highest (Krippendorff’s alpha (0.47).

## Discussion

GRE-9, the new rubric for evaluating reflection in medical education, is a short, easy to use, and reliable assessment tool. The items of this rubric were based in theory and were clearly presented. A distinguishing feature of the GRE-9 is that its 8th item: “Reference to old experience and to others” was explicitly presented in the tool unlike the REFLECT rubric. The explicit reporting of this item was in accordance with Mezirow’s [19] theoretical underpinnings of reflection that focus on recognition and expression of the internal states and conditions of others and attitudes held towards them. The item was also in accordance with Schon’s [2] documentation that in professional work reflectivity focuses on social observation, interaction, and meaning given to various interactions. This delineated item reflects relevance for gaining insight into the cultural context of Lebanon. The collectivistic culture of Lebanon allows one to emphasize on the significance of the other and the interactions formed with them [43]. Hence, although the GRE-9’s theoretical framework was based on theoretical underpinnings formulated from the West and despite the notion that reflective assessment tools can be used globally irrespective of rater contextual and education background [44], the cultural relevance of the 8th item renders an advantage to the GRE-9 and its applicability in a specified cultural context.

The REFLECT rubric was a starting point for the development of the GRE-9 items. The precedence of REFLECT rubric in comparison to the GRE-9 lies in its already obtained validity evidence in similar contexts of measuring medical students’ reflective capacity [17, 45] as well as in REFLECT’s slightly higher inter-rater reliability obtained in previous studies compared to GRE-9 [15, 17]. The REFLECT rubric can also be regarded as more elaborative as it incorporates three additional grading levels: “Writing Spectrum i.e. exploration and critique of assumptions, values, beliefs, and/or biases, and the consequences of action (present and future)”, “Presence i.e. sense of writer being fully present” and “Attention to assignment i.e. whether the writer answers the assignment question or, if relevant provides a compelling rationale for choosing an alternative” [17]. As such, the REFLECT does not only measure the reflective ability but also presents input on the extent to which the student was involved and engaged in the reflective and writing process; thus, providing richer information with regard to the credibility of what was reported and written by the medical student as well as the student’s reflection ability. These grading levels are not present in the GRE-9. However, the advantage of the GRE-9 in comparison to the REFLECT rubric lies in the GRE-9’s item simplicity and clarity in comparison to what was reported with regard to the REFLECT rubrics’ needed revision on item clarity [15]. Also, while the REFLECT rubric is designated as a formative rubric lacking grading [17], the formative and summative nature of the GRE-9 encourages the analysis of the quality of reflection as well as the assignment of numbers to the reflection levels as an anchor on which discussing the outcomes of student’s reflection level can be explored. This is in line with the call in research for the need to incorporate quantitative and summative reflective assessment rubrics into the learning processes [46].

It has been reported that reflective tools founded on previous work of reflective assessment and theoretical frameworks provide the premise for different raters to reach same interpretive results when assessing reflection [17]. Literature has revealed that rubrics can be utilized internationally across diverse educators, cultural backgrounds and education curricula [6, 44]. For instance, results of a study conducted by Lucas et al. [44] revealed that the reflective rubric for assessing reflective essays of pharmacy students that was used by three different raters from different educational backgrounds and cultural contexts maintained a high rater agreement; thus, indicating that the rubric they used is a reliable tool capable of being applied across different educational settings irrespective of the contexts and/or different educational curricula. Consequently, it can be fathomed that the GRE-9 will not only be useful within the AUBMC context, but it can also be presented into the literature of medical education for assessing reflective essays by others professionals.

The results of this study yielded a moderate to substantial inter-rater reliability for the GRE-9 based on the ICC and krippendorff’s alpha. In fact, the inter-rater reliability of the GRE-9 scale (ICC of 0.78) is considered good in comparison to previous studies; for instance, it was only slightly lower than the inter-rater reliability reported by Lucas et al. [6] who found an average measure ICC of 0.81 for their own developed reflective rubric to assess pharmacy students’ reflective thinking as well as slightly lower than the five-rater ICC (alpha) of 0.80 for the REFLECT rubric composite of reflective written essays of medical students [45].

The lack of inter-rater agreement on item 2 “What is special about this event- State clearly the reason for choosing this event in particular” might be attributed to the role of subjectivity arising when rating this item. The reason behind choosing to report about a particular event in the reflective essay might not be stated explicitly, but rather implied. Hence, subjectivity and variability among the raters when assessing the implied reason might arise. The reason behind the low inter-rater agreement for item 5 “Understanding of the event - Express what was good and bad about the experience; interpretation of the situation at present with justifications (factors/ knowledge influencing judgment)” could be the difficulty in detecting a clear and full description of the disorienting dilemmas, issues of concern incorporating multiple perspectives, alternative explanations and challenging assumptions especially within written essays especially that identical criterion phrasings might have been used when reporting the different levels of description of conflict and its conclusions. The high inter-rater agreement for item 3 “Feelings when it happened - Describe personal thoughts and feelings of the resident while the event was happening” (attending to feelings) can be explained by the fact that an important aspect of reflection is pointing and dwelling on emotions [2]; hence, residents might have profoundly focused on expressing their emotions in their reflective writings explicitly and clearly allowing the raters to detect emotions and feelings easily.

In a previous study that aimed to investigate the reliability, feasibility, and responsiveness of a categorization scheme for assessing pharmacy students’ levels of reflection [12], the mean timing that was needed for only categorizing one essay was 3 min; this time measured was only for the grading procedure and was reported to be prone to increase if used in formative assessments. It was also considered a reasonable timing based on the feasibility test [12]. The time taken to grade using the GRAS was around 10 min [1]. Hence, the time needed to read and grade the reflective essays in this paper shows that GRE-9 seems to be a reasonably fast method in assessing the level of reflection in the written reflective essays. This reasonable timing will be especially important when used in teaching settings [12].

Although the new instrument’s validity was not tested empirically, it is vital to note that theoretically, the newly developed scale incorporates theoretical themes that match with the themes that emerged in validated instruments measuring reflection of medical students. For instance, and as stated previously, the reflection themes in the newly developed instrument match with the thematic structure of the “Reflection Evaluation for Learners’ Enhanced Competencies Tool (REFLECT)” [17]. Similarly, the thematic underpinnings of the newly developed scale overlap with the three emerged thematic factors of personal reflection: self-reflection, empathetic reflection and reflective communication of the “Groningen Reflection Ability Scale (GRAS)” [1]. For instance, the items “ (1) What happened (2) What is special about this event (3) Feelings when it happened (5) Understanding of the event (6) Congruence of actions and beliefs of the newly developed scale are compatible with the self-reflection criterion. Items: (4) What was the outcome for the concerned (7) New thoughts and feelings after reflection are compatible with the empathetic reflection criterion, and items: (8) reference to old experience and others (9) How this incident will affect future role, are consistent with the reflective communication criterion which integrates reflective behavior, openness for feedback and discussion, taking responsibility for own statements, actions and ethical accountability [1]. Consequently, given that the items of the GRE-9 scale were conceptually and thematically based on solid theoretical underpinnings and match with the three essential aspects of personal reflection in the context of medical practice and education of the GRAS tool as well as match with the four reflective levels of the REFLECT tool, the content validity of the scale can be assumed.

The moderate to substantial inter-rater reliability of the GRE-9 allows raters to objectively grade reflective essays as well as provide informed feedback to residents and students. It is important to note however that developing a reliable reflective tool alone does not seem to be the sole solution in solving the problem of rater variability and fair assessment of reflective capability. Solutions for unfairness and variability can be reached by structurally aligning raters on the interpretation of the reflective rubrics through robust rater-training programs [12], by increasing the number of observations for increased accuracy of reflection assessment [5] and by assessing multiple reflective samples per students so as to produce a significant conclusion about the reflective capacity [5].

A number of limitations can be pointed to in this paper. Primarily, this study was conducted solely at AUBMC which might limit the generalizability of the results. Also, this study evaluated single reflective-writing samples per students; hence, this too might limit the generalizability of the results because reflective writings are considered to be context-dependent and many skills can be assessed in medical education, so a single writing sample might not provide an accurate estimate of the reflective competency of students [5]. Future research with multiple writings will be needed to test the rubric’s psychometrics even further [6]. Also, the sample size was not enough to calculate construct validity which have influenced the obtainment of rigorous psychometric properties for the scale. Given that the reflective essays were an assignment asked for from Family Medicine residents during their 3 years of training, performance bias might have impacted the write-up of the reflective essays as they sought approval from their advisors or peers [47].

## Conclusion

GRE-9 is a reliable, concise, simple grading tool that has demonstrated moderate to substantial inter-rater reliability that will enable raters to objectively grade reflective essays and provide informed feedback to residents and students. Future research will need to investigate the validity of the scale empirically such as exploring the external validity by comparing the scores of this instrument with other reflection scales and measures of reflective practice outcomes as well as to use a larger sample so as to ensure a more rigorous psychometric soundness of the scale.

## Supplementary information

**Additional file 1.**

## Data Availability

The datasets used and/or analyzed during the current study are available from the corresponding author on reasonable request.

## Abbreviations

AUBMC: American University of Beirut-Medical Center
ICC: Intra-class correlation coefficients
IRB: Institutional Research Board
IRR: Inter-rater reliability
GRE: Grading Reflective Essays
