# Supplementary material for: Grading reflective essays: the reliability of a newly developed tool- GRE-9
Source: BMC Med Educ. 2020 Sep 25;20:331. doi: 10.1186/s12909-020-02213-2 (PMC7520967; doi:10.1186/s12909-020-02213-2)

Appendix I

**Assessment of Reflective Essays**

Ask the resident to tell you a case from her/his practice that made her/him reflect on it.

Below is a guide to assess reflective essays. Indicate your assessment by giving a grade from 0-3.


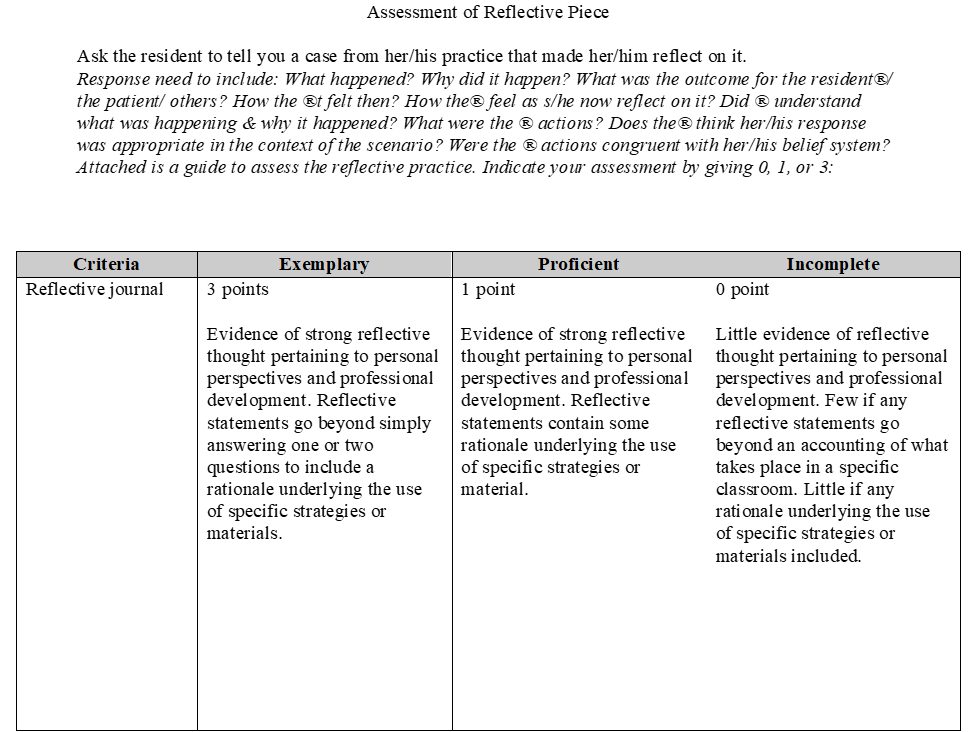

Supplement: Supplementary file 1 — Additional file 1. [file 12909_2020_2213_MOESM1_ESM.docx]
